# Screening Algal and Cyanobacterial Extracts to Identify Potential Substitutes for Fetal Bovine Serum in Cellular Meat Cultivation

**DOI:** 10.3390/foods13233741

**Published:** 2024-11-22

**Authors:** Nikolina Sibinčić, Maja Krstić Ristivojević, Nikola Gligorijević, Luka Veličković, Katarina Ćulafić, Zorana Jovanović, Aleksandar Ivanov, Lora Tubić, Carole Vialleix, Thibaut Michel, Tatjana Srdić Rajić, Milan Nikolić, Marija Stojadinović, Simeon Minić

**Affiliations:** 1 Innovative Centre, University of Belgrade-Faculty of Chemistry, 11000 Belgrade, Serbia; nsibincic@chem.bg.ac.rs; 2 Department of Biochemistry & Center of Excellence for Molecular Food Sciences, University of Belgrade-Faculty of Chemistry, 11000 Belgrade, Serbia; krstic_maja@chem.bg.ac.rs (M.K.R.); aleksandar_ivanov2001@hotmail.com (A.I.); sminic@chem.bg.ac.rs (S.M.); 3 Department of Chemistry, Institute of Chemistry, Technology, and Metallurgy, National Institute of the Republic of Serbia, University of Belgrade, 11000 Belgrade, Serbia; 4 GreenSea, 34140 Mèze, France; 5 Institute for Oncology and Radiology of Serbia, 11000 Belgrade, Serbia

**Keywords:** algae, cyanobacteria, fetal bovine serum, alternative, cell culture, cultivated meat

## Abstract

Cultured meat technology is a form of cellular agriculture where meat is produced from animal cells grown in a lab, instead of raising and slaughtering animals. This technology relies heavily on fetal bovine serum (FBS) in cell media; hence, production is costly and contributes significantly to ammonia and greenhouse gas emissions. Achieving the successful commercialization of cell-cultured food requires the critical resolution of manufacturing cost and safety concerns. Hence, our research efforts are focused on identifying commercially viable and ecologically sustainable alternatives to FBS. In this study, we evaluated the potential of twenty-six water-based algal and cyanobacterial extracts to stimulate cell growth for meat cultivation under 90% reduced serum conditions. The extracts were compared in viability, proliferation, and Trypan blue exclusion assays. In the first screening phase, the extracts were evaluated in a ZEM2S (zebrafish) cell culture in a 1% FBS regimen. Based on their ability to exhibit protein tolerance or promote cell proliferation, ten extracts were selected and further assayed in a QM7 cell culture. The QM7 cell line (myoblasts from Japanese quail) is highly relevant for meat cultivation because of its ability to differentiate into muscle fibers. Extracts derived from two microalgae species, *Arthrospira platensis* (*Spirulina*) and *Dunaliella tertiolecta*, demonstrated the highest tolerance in cell culture, above 10 μg/mL (expressed as total protein concentration). Tolerance at a 100 μg/mL concentration was demonstrated exclusively using an extract of blue spirulina (commercially purified *Spirulina*), which supported cell growth through multiple passages.

## 1. Introduction

Meat represents an important component of the human diet. Considering the population increase, which is predicted to reach 10 billion in 2050, there will be a corresponding rise in the demand for meat. Over 300 million tons of meat were consumed in 2014, predicted to increase by 76% by 2050 [1]. Based on animal husbandry, traditional meat production has several emerging sustainability problems. Using the traditional approach, animal production should increase to keep up with the growing population and demand for meat. Due to animal husbandry, more arable land will be occupied, more fresh water will be used, and greenhouse gas (GHG) emissions will further increase [2]. This problem raises issues regarding the sustainability of this approach and requires significant transformation rather than just minor adjustments [3]. Additional problems regarding traditional meat usage are moral issues connected to animal slaughter, the contribution of animal meat to cardiovascular problems and animal food-borne diseases, and the development of antibiotic-resistant bacterial strains [4,5,6].

Meat alternatives include plant proteins or microbial fermentation products with similar aesthetic qualities to specific types of meat [7]. Another concept that is gaining increasing attention is cultivated or in vitro meat. It is advantageous to plant-based and fermentation-derived meat products because cultivated meat products are the closest to animal meat products in terms of their biochemical composition, taste, appearance, and texture. Cultivated meat, a concept that first appeared in a scientific novel in 1897, did not develop much interest until the end of the 20th century. From 2013 onwards, the number of studies on cultivated meat rapidly increased. With the possibility of the future introduction of cultivated meat in the food industry and on the market, scientific research must continue to address all the current drawbacks regarding the broader usage of cultivated meat [8]. Cultivated meat offers several advantages over traditionally produced meat, including functional and designer meat, animal welfare, reductions in zoonotic and food-borne diseases, quick production, reduced resource utilization, ecological benefits, vegan meat, etc. [9]. It has been estimated that if cyanobacteria are used as an energy and nutritional source for cell culture media, the production of 1000 kg of cultured meat would require 7–45% less energy, 82–96% lower water usage, and 99% lower land usage, with 78–96% lower GHG emissions, compared to the same amount of meat if traditionally produced [10]. 

The price of cultured meat mainly depends on the cell media, which provide essential nutrients and growth factors to support cell proliferation and differentiation. Fetal bovine serum (FBS) is often used in media to provide essential nutrients, hormones, and growth factors. It is the major cost contributor and has several issues, such as inconsistency, contamination risks, and ethical issues [3]. To produce 0.5 million liters of FBS, more than one million fetuses need to be slaughtered [11]. In this context, one of the most significant challenges in the cultivated meat field is the development of serum-free media to ensure sustainability and scalability. Replacement can be achieved by the utilization of recombinant albumin together with growth factors [12]. However, this approach has yet to be considered cost-effective. Hence, partial or total albumin replacement strategies must be developed [13]. Extracts and hydrolysates, rich in nutraceuticals, are good candidates to ultimately or significantly reduce the usage of FBS. They should be obtainable from cheap sources to decrease the high price of cultured meat production. In this regard, biomass derived from algae and cyanobacteria is very attractive and has great potential. Algae and cyanobacteria represent good alternative sources of nutrients, like proteins, peptides, amino acids, sugars, fats, minerals, and vitamins [14]. The additional advantage is that using algal biomass enables the coupling of cultured meat production with other sustainable processes like CO_2_ fixation and waste media recycling [15]. Ideas about the circular growth of algae and mammalian cell cultures are also emerging, where one medium is used back and forth, with proper adjustments [13,16]. However, only a few studies have tackled the development of algal- and cyanobacterial-based products as FBS alternatives for sustainable meat cultivation [17,18,19].

In this study, a screening platform was developed to test the potential of algal and cyanobacterial extracts to stimulate cell growth for meat cultivation under reduced serum conditions. First, cell viability and proliferation assays were performed to test the twenty-six algal and cyanobacterial extracts on the ZEM2S cell line (fibroblasts established from zebrafish embryos) within a short-term time scale. The ten extracts with the highest activity were further tested on the same cell line, ZEM2S cells, and the QM7 cell line (myoblasts from Japanese quail), which is highly relevant for meat cultivation, using both short-term and semi-long-term cultivation approaches. Finally, the correlation between the biochemical composition of the extracts and their potency to stimulate cell growth at reduced serum conditions was investigated. 

## 2. Materials and Methods

### 2.1. Reagents and Consumables

Fetal bovine serum of South American origin (cat. no. 10270106) and tryptose phosphate broth (TPB, 18050039) were purchased from Gibco (Grand Island, NY, USA). Galic acid was purchased from the Tokyo Chemical Industry (Tokyo, Japan). Sulfuric acid was purchased from Centrohem (Stara Pazova, Serbia). L-15 Medium Leibovitz (L1518), Dulbecco’s Modified Eagle’s Medium high glucose (D5648), Nutrient Mixture F-12 Ham (N6760), Medium 199 (M4530), 100× L-glutamine (G7513), 100× penicillin–streptomycin (P07B1), 1 M HEPES buffer solution (H0887), dimethyl sulfoxide (DMSO), 3-(4,5-dimethylthiazol-2-yl)-2,5-diphenyltetrazolium bromide (MTT), 7-hydroxy-3*H*-phenoxazin-3-one-10-oxide (resazurin, Alamar Blue, AB), 3-amino-7-dimethylamino-2-methylphenazine hydrochloride (neutral red, NR), Trypan blue, 0.25% trypsin–EDTA, sodium acetate, sodium bicarbonate, hydrochloric acid, Folin–Ciocalteu’s phenol reagent, glucose, 2,4,6-tris (2-pyridyl)-s-triazine (TPTZ), ferric chloride hexahydrate, 6-hydroxy-2,5,7,8-tetramethylchroman-2-carboxylic acid (Trolox), 5-bromo-2′-deoxyuridine (BrdU, B5002), mouse monoclonal anti-BrdU antibody (B8434), goat anti-mouse IgG-AP antibody (A3562), *p*-nitrophenyl phosphate, and all other chemicals were purchased from Sigma-Aldrich, Merck (Darmstadt, Germany). Cells were maintained in T-75 and T-25 BioLite™ tissue culture-treated flasks with filter caps from Thermo Fisher Scientific, Inc., Waltham, MA, USA. Cell-based experiments were performed in clear, flat-bottom, 96-well BioLite™Plastic sterile labware (Thermo Fisher Scientific, Inc.).

### 2.2. Preparation and Characterization of Extracts

#### 2.2.1. Preparation of Extracts

Most algae and cyanobacteria were cultivated by Green Sea, Mèze, France, and provided to the laboratory in their lyophilized form. *Arthrospira platensis* (Marcus Rohrer, Padova, Italy), blue spirulina (We Are One, Sombor, Serbia), and *Porphyra yezoensis* (RawNori, Palmdale, CA, USA) were commercially available. In total, twenty-six extracts were prepared by extracting the dry biomass with 5 mM phosphate buffer pH 8.0 using a magnetic stirrer for 4 h (1 g of dry biomass per 20 mL of buffer). Then, the obtained suspension was centrifuged at 10,000× *g*, 4 °C for 30 min. The *Nannochloropsis gaditana* extract was prepared by extracting the alga with 10 mM NaOH in alkaline conditions, followed by pH neutralization with HCl. All extracts were filtered through sterile 0.22 µm membrane syringe filters in a class II biosafety cabinet and stored at −80 °C as single-use aliquots. The protein concentration was determined in a Bradford assay, using BSA as a standard. 

#### 2.2.2. Biochemical Analysis of Extracts

The total phenolic content in the extracts (*n* = 2, diluted with MiliQ water five times) was determined according to Folin–Ciocalteu’s method, adopted to microtiter plates using gallic acid as a standard [20]. The total sugars in the extracts (*n* = 2, diluted 20–800 times, depending on the extract) were determined using the phenol–sulfuric acid method, according to the published protocols and adapted to the microtiter plates using glucose as a standard [21]. The glucose concentration in the extracts (*n* = 2, diluted 5–200 times, depending on the extract) was determined using a commercial glucose assay kit GAG020-1KT (Merck, Darmstadt, Germany), following the manufacturer’s instructions. 

#### 2.2.3. Ferric-Reducing Antioxidant Power (FRAP) Assay

The antioxidant capacity of the algal extracts was measured by the FRAP assay with minor modifications to the protocol of Subbiah et al. (2021) [22]. The FRAP reagent was prepared by mixing 12.5 mL of 300 mM sodium acetate buffer at pH 3.6, 1.25 mL of 10 mM TPTZ diluted in 40 mM HCl, and 1.25 mL of 20 mM ferric chloride hexahydrate (reagent amount sufficient for one 96-well plate). Based on the concentrations of algal proteins, the samples were diluted in phosphate-buffered saline (PBS) at ratios of 1:2, 1:5, or 1:10. The test was performed in a 96-well plate in triplicate, and the sample to working solution ratio was 1:20 (*v*/*v*). The mixture was incubated in the dark at ambient temperature for 30 min. The absorbance of the samples was measured at 593 nm on a BioTek Synergy H1 multimode plate reader (BioTek Instruments, Winooski, VT, USA). The antioxidant capacity (AC) was determined from the Trolox standard curve and expressed as µmol Trolox equivalents per mg of protein (calculated by dividing the Trolox µmol/mL values read from the standard curve with the concentration of proteins expressed in mg/mL).

### 2.3. Cell Lines and Culturing Conditions

Zebrafish embryonic fibroblasts (ZEM2S) (CRL-2147) and quail muscle myoblasts (QM7) (CRL-1962) were obtained from the American Type Culture Collection (ATCC) (Manassas, VA, USA). ZEM2S were cultured in a medium composed of 50% L-15, 35% DMEM, and 15% Ham’s F12 supplemented with 10% (*v*/*v*) heat-inactivated FBS, 0.18 g/L sodium bicarbonate, 15 mM HEPES, and 1% (*v*/*v*) penicillin–streptomycin at 28 °C in a CO_2_-free environment. ZEM2S cells were used within passages 12 to 25. QM7 cells were maintained in Medium 199 with Earle’s BSS supplemented with 10% (*v*/*v*) FBS, 10% (*v*/*v*) TPB, and penicillin–streptomycin at 37 °C in a humidified 5% CO_2_ atmosphere. QM7 cells were used between passages 15 and 24. 

### 2.4. Cell Viability Assays

#### 2.4.1. MTT Assay

The MTT assay was performed according to a slightly modified procedure derived from Mosmann, 1983 [23], on QM7 cells seeded at 10,000 cells/well in 200 µL of complete media. The next day, the media were replaced with 200 µL of 1% FBS-reduced serum media with or without extracts in the protein concentration range of 0–10 µg/mL. After 72 h of incubation, 20 µL of 5 mg/mL MTT–PBS solution was added to each well and they were incubated at 37 °C and 5% CO_2_ for ~2 h. The media were discarded, and the formed MTT formazan crystals were dissolved in 150 µL DMSO. The absorbance was read at 570 nm. The viability of the treated cells was calculated by normalizing the sample absorbance to the untreated cells. Samples were prepared in triplicate, including the corresponding controls. 

#### 2.4.2. Alamar Blue/Resazurin Assay

The Alamar Blue (AB) assay was performed on ZEM2S cells seeded at 15.000 cells/well in 200 µL of 1% FBS-reduced media with algal extracts in the protein concentration range of 0–100 µg/mL. After 72 h of incubation, the cells were spun at 500 g for 10 min, the media were discarded, and 100 µL of 0.03 mg/mL resazurin medium solution without serum was added to each well, and they were incubated at 28 °C in a CO_2_-free atmosphere for ~3–4 h. After incubation, the media were transferred to fluorescence-compatible 96-well plates, and the pink resorufin fluorescence was measured with a filter set configured at 530/25 nm for excitation and 590/35 nm for emission. The viability of the treated cells was determined by normalizing the absorbance of the samples to that of the untreated cells. The assay was performed in triplicate for each set of conditions, including the corresponding controls. The resazurin assay was adapted from the Kumar et al. (2018) protocol [24].

#### 2.4.3. Neutral Red Uptake Assay

The neutral red uptake (NRU) assay was conducted on ZEM2S cells, with the experimental setup following the seeding protocol previously described for the resazurin assay. After 72 h of cell incubation with algal extracts in 1% FBS-reduced media, the media were discarded, and 100 µL of 5 µg/mL neutral red (NR) working solution, prepared the day before in media without serum, was added to each well, and they were incubated at 28 °C in a CO_2_-free atmosphere for ~3–4 h. After incubation, the cells were spun at 500 g for 10 min; the NR solution was discarded. The cells were gently washed with 150 µL of PBS and spun at 500 g for 10 min. After discarding the PBS, the NR dye was extracted by adding 150 µL of extraction (destain) solution (50% (*v*/*v*) ethanol, 1% (*v*/*v*) glacial acetic acid) to each well. The microplate was incubated on a plate shaker for at least 10 min or until the NR had been extracted from the cells and formed a homogeneous solution. The destaining solution with extracted dye was transferred into fluorescence plates (Sarstedt, Nümbrecht, Germany), and the NR fluorescence was measured on a BioTek multimode plate reader with the excitation/emission filter set configured at 530/25 nm for excitation and 590/35 nm for emission. The viability of the treated cells was determined by normalizing the absorbance of the samples to that of the untreated cells. The assay was performed in triplicate for each set of conditions, including the corresponding controls. The NRU assay was optimized using the Repetto et al. (2008) protocol, with minor modifications [25].

### 2.5. Bromodeoxyuridine Proliferation Assay

ZEM2S and QM7 cells were seeded as per the previously established viability protocols, with each condition, in triplicate. The following day, 2 µL of BrdU (100× stock, dissolved 40 mg/mL at 50 °C) was added per well. Forty-eight hours later, the cell plates were centrifuged at 500 g for 10 min, and the old media were discarded. Cells were washed with PBS, spun, and fixed for 30 min using 200 µL of 3.7% formaldehyde in PBS. After two additional PBS washes followed by centrifugation, the cells were permeabilized for 30 min with 200 µL of 0.1% Triton X-100 in PBS. The washing procedure was repeated twice with PBS. To denature the DNA and make the BrdU accessible to the antibody, 200 µL of 2 M HCl was added per well and they were incubated for 30 min. The cells were washed three times with 0.1% Triton X-100-PBS and blocked for one hour with 200 µL of 1% BSA in 0.1% Triton X-100-PBS. After two additional washes with 0.1% Triton X-100-PBS, 50 µL of anti-BrdU antibody diluted 1:1000 in blocking buffer was added to each well and they were incubated overnight at room temperature. The next day, the plates were washed four times with 0.1% Triton X-100-PBS, and 100 µL of goat anti-mouse IgG-AP diluted 1:10,000 in blocking buffer was added for 1–2 h of incubation. After three washes with 0.1% Triton X-100-PBS and one wash with PBS, 100 µL of 1 mg/mL *p*-nitrophenyl phosphate substrate (1.4 mg/mL; hexahydrate) in AP ELISA buffer (10 mM diethanolamine, 0.5 mM MgCl_2_, pH 9.8) was added. Absorbance was measured at 405 nm, with color development observed within 30 min. The BrdU ELISA protocol was optimized following the manufacturer’s guidelines. 

### 2.6. Trypan Blue Exclusion Assay 

ZEM2S cells were seeded in 48-well plates at a density of 50.000 cells per well in 400 µL of 1% FBS-reduced media supplemented with algal extracts in duplicate. The cells were incubated for three days, with the morphology monitored daily (after 24, 48, and 72 h). On the third day, the Trypan blue (TB) exclusion test was performed. The cells were spun at 500× *g* for 10 min before trypsinization to prevent potential cell loss. The old media were discarded, and the cells were detached with 100 µL of 0.25% trypsin–EDTA. The enzyme was inactivated with 400 µL of fresh media, and the cells were spun at 500× *g* for 10 min. The media were disposed of; the cells were resuspended in 100 µL of media and mixed in a 1:1 (*v*/*v*) ratio with 0.4% (*w*/*v*) TB solution in PBS. The cells were counted in all fields of the dual-chamber hemocytometer (Neubauer, Germany) under a light microscope. In all samples, the cell viability was 95% or higher. The viability of the treated cells was normalized to that of the control. 

### 2.7. Cell Adaption to Low-Serum (1% FBS) Media Supplemented with Blue Spirulina 

Blue spirulina extract was selected for the ~1 month maintenance of ZEM2S and QM7 cells in 1% FBS-reduced media at 10 and 100 µg/mL protein concentrations in a T-25 flask. Control cells in 1% FBS were also included. Cell counting was conducted for the first three passages for QM7 and the first four passages for ZEM2S using TB. The passaging of the cells included common steps, as described in the previous section. QM7 cells underwent six passages over approximately one month, with subculturing occurring every four days at 2E5 cells/T-25 flask. Following the fourth passage, the QM7 cells supplemented with 100 µg/mL of extract were discarded. In the case of ZEM2S cells, both concentrations were maintained throughout the entire duration of monitoring (six passages). These cells were subcultured on the fourth day during the first two passages with 8E5 cells/T-25 flask. However, due to the observed slow proliferation, subsequent passages were conducted every sixth day at the 1E6/T-25 flask, with media changes every third day. Cell confluency was also monitored and measured during this time by the Millicell^®^ Digital Cell Imager MDCI10000 (Merck Millipore, Darmstadt, Germany). The spent cell media were preserved for subsequent pH measurements.

### 2.8. Statistical Analysis

One-way ANOVA and Dunnett’s multiple comparisons test were used to compare the different concentrations of algal extracts to the corresponding controls—the untreated cells. The GraphPad Prism 7.0 software (GraphPad, San Diego, CA, USA) was used to create the figures and perform the statistical analysis.

## 3. Results

### 3.1. Primary Screening of Algal/Cyanobacterial Extracts in ZEM2S Fibroblasts—Testing Extracts’ Cytotoxicity

The primary screening’s goal was to determine which algae/cyanobacteria, and which concentrations, have a beneficial effect in promoting ZEM2S cell viability. As most of the FBS is albumin, our study focused on the protein component of the extracts. Therefore, we used the protein concentration to quantify the amount of the extracts added to the cells. ZEM2S cells were routinely maintained in 10% FBS-supplemented basal media (full composition in Material and Methods). On the day of the experiment, the cells were passaged and seeded in 96-well plates in 1% FBS-supplemented media (90% reduction in FBS) with or without algal/cyanobacterial extracts. The usual seeding procedure, where cells are seeded in 10% FBS and the treatments are added the next day, could not be implemented for ZEM2S cells as, in our experience, they partially detach from the bottom of the plate during media exchange. Cells were incubated with the extracts for 72 h, rather than the usual 24 h used in general cytotoxicity assays, because we were looking for candidates with long-term cell tolerance. The resazurin reduction assay, also known as the Alamar Blue (AB) cytotoxicity assay, was chosen over the MTT assay as the ZEM2S cells did not metabolize the tetrazolium salts in our hands, and AB offered higher sensitivity due to the fluorescence read-out.

Twenty-six extracts were tested in total, including seven from cyanobacteria (two formulations of *Arthrospira platensis*, *Anabaena ineaqualis*, *Nostoc* species, *Pseudanabaena galeata*, *Chroococcidiopsis thermalis*, *Synechococcus nidulans*), four from *Rhodophyta* (*Porphyridium aerugineum*, *Rhodella maculata*, *Porphyra yezoensis*, *Porphyridium purpureum*), eight from *Chlorophyta* (*Chlamydomonas nivalis*, *Chlamydomonas renhardtii*, *Chlorella vulgaris*, *Dunaliella salina*, *Dunaliella tertiolecta*, *Haematococcus pluvialis*, *Tetraselmis suecica*, *Selenastrum capricornutum*), five from *Heterokontophyta* (*Xanthonema species*, *Nannochloropsis gaditana*, *Nannochloropsis oculata*, *Cylindrotheca closterium*, *Phaeodactylum tricornutum*), and two from *Haptophyta* (*Isochrysis galbana*, *Diacronema lutheri*). 

**Figure 1:**
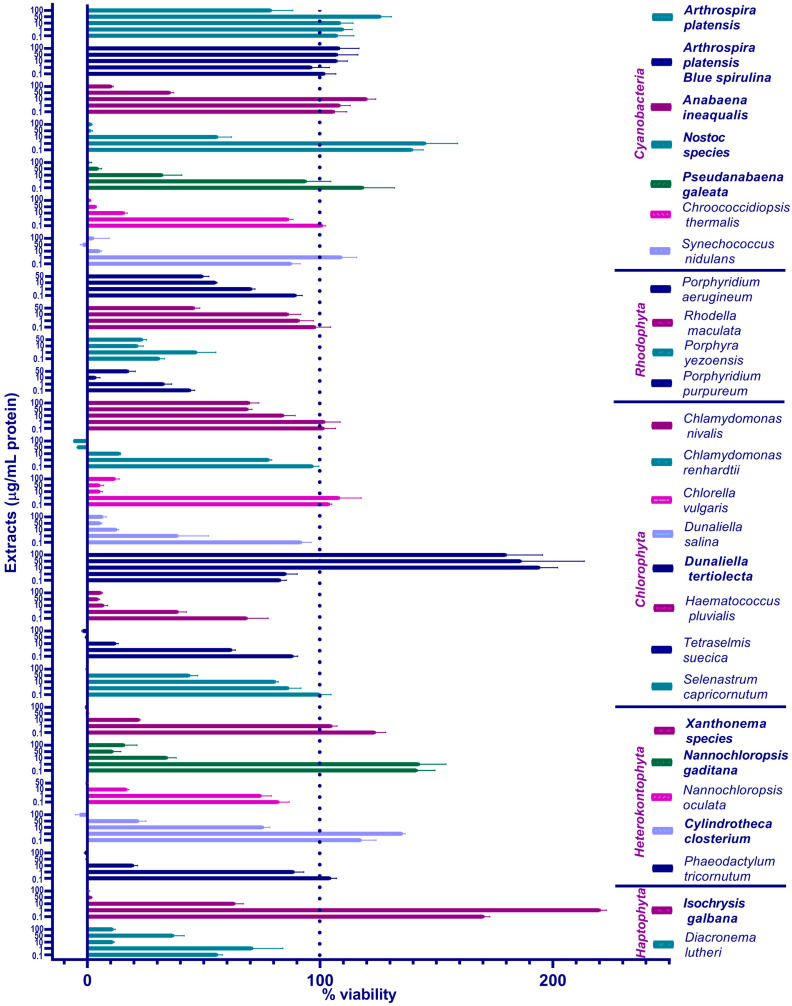
We screened 26 algal/cyanobacterial extracts to determine their impact on the viability of ZEM2S cells. Cell viability was estimated in the Alamar Blue (resazurin) assay after 72 h of treatment and normalized to the non-treated/control cells, which were at 100%. Extracts were tested at 0.1, 1, 10, and 50 µg of protein per mL and, where possible, at 100 µg/mL. Each extract was tested at least twice; the graph presents the mean and SEM values of triplicate runs from one representative experiment. The legend contains the full names of the species, color-coded to match the columns and organized according to their phylum affiliation (for strain taxonomy, we used AlgaeBase: https://www.algaebase.org, accessed on 16 September 2024). Extracts selected for the second round of screening are shown in bold letters.

Figure 1 shows the results of the primary screening obtained by the AB assay: the mean values with standard errors (SEM) of the percent viability in the presence of different concentrations of the extracts. The general trend observed in all algae/cyanobacteria was a reduction in cell viability at concentrations ≥ 10 μg/mL, especially at 50 and 100 μg/mL. Nevertheless, the effect of *Arthrospira platensis* and *Dunaliella tertiolecta* on cell viability was superior even at higher concentrations of 50 and 100 μg/mL. The phycocyanin-enriched extract of blue spirulina was better tolerated at 100 μg/mL than the full cyanobacterial extract labeled as *Arthrospira platensis*. On the other hand, the alga *Dunaliella tertiolecta* proved to be very interesting in the context of cell survival due to the inverse trend shown—increasing concentrations enhanced the cell viability.

The condition for the extract to move to the second round of viability testing was as follows: algal tolerance at a concentration equal to or greater than 10 μg/mL or an enhancement in cell viability at any concentration. This condition was met by ten extracts: five from cyanobacteria, including *Arthrospira platensis*, blue spirulina, *Anabaena ineaqualis*, the *Nostoc* species, and *Pseudanabaena galeata*, one from *Chlorophyta* (*Dunaliella tertiolecta*), three from *Heterokontophyta* (the *Xanthonema* species, *Cylindrotheca closterium*, and *Nannochloropsis gaditana*), and one from *Haptophyta* (*Isochrysis galbana*).

**Figure 2:**
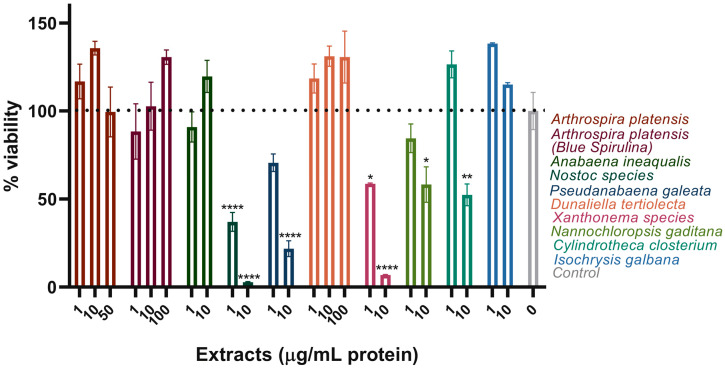
Cytotoxicity of ten selected algal/cyanobacterial extracts on ZEM2S cells was measured by the neutral red uptake assay after 72 h of treatment and normalized to the non-treated/control cells, which was at 100%. Each extract was tested at least twice; mean and SEM values of triplicate runs from one representative experiment are presented on the graph. The legend contains the full names of the species, color-coded to match the columns. Statistically significant differences between the control and the treatment groups are labeled with * (*p* ≤ 0.05), ** (*p* ≤ 0.01), or **** (*p* ≤ 0.0001).

The ZEM2S cells were treated with the ten selected extracts at a few concentrations around the optimal values obtained from the AB assay. Cell health was assessed using the NRU assay. The NRU assay is based on the ability of viable cells to incorporate and bind the supravital NR dye within lysosomes. Both AB and NRU are useful assays to combine, as they measure different parameters (cellular redox potential vs. lysosomal integrity) and, in conjunction, can provide a more realistic outcome. *A. platensis* and *D. tertiolecta* were once again tolerated by the cells without any significant loss in cell viability, even at the highest tested concentration; some boost in cell viability was also observed (Figure 2). As in the AB assay, *A. ineaqualis* and *I. galbana* were not toxic at 1–10 μg/mL, but *Nostoc* spp., *P. galeata*, *Xanthonema* spp., and *N. gaditana* did not perform as well as in the AB assay. *C. closterium* did not impair NR uptake only at the lowest tested concentration of 1 μg/mL (Figure 2). Large boosts in cell viability were observed in the AB assay, especially for *I. galbana* and *D. tertiolecta*, but they were not as pronounced in NRU. 

### 3.2. Secondary Screening of the Selected Extracts in ZEM2S Fibroblasts—Examining the Effect on Cell Proliferation

**Figure 3:**
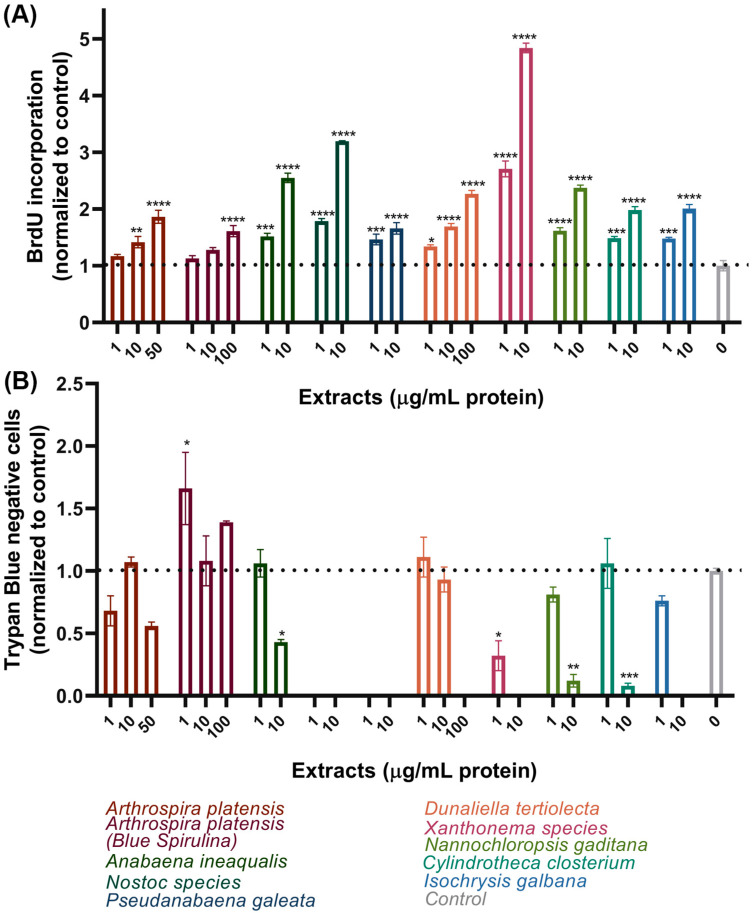
The effect of the selected extracts on ZEM2S proliferation. Cell proliferation was assessed by measuring DNA synthesis in a BrdU incorporation assay at 48 h (**A**) and by counting cells in a Trypan blue exclusion assay at 72 h (**B**). Each extract was tested at least twice; mean and SEM values of triplicate runs from one representative experiment are presented on the graph. The legend contains the full names of the species, color-coded to match the columns. Statistically significant differences between the control and the treatment groups are labeled with * (*p* ≤ 0.05), ** (*p* ≤ 0.01), *** (*p* ≤ 0.001), or **** (*p* ≤ 0.0001).

The effect of the extracts on cell progression through the later stages of the cell cycle, the S-phase of the interphase, and mitosis was assessed by measuring the level of DNA synthesis and counting the living cells at the end of the treatment (Figure 3). DNA synthesis was quantified and compared to the control using the BrdU enzyme-linked immunosorbent assay (ELISA). This technique involves incorporating a synthetic thymidine analog, BrdU, into newly synthesized DNA strands, which is then detected using specific antibodies in an ELISA format. As ZEM2S cells do not divide rapidly, BrdU was added at 24 h after seeding with the extracts in 1% FBS media and kept throughout the next 24 h or until 48 h after the beginning of the experiment. The results were normalized to the untreated control, as presented in Figure 3A. *A. platensis* in both extract and purified form (blue spirulina) did not significantly affect DNA synthesis (fold change of 1.2–1.4 when compared to the control), except at the highest tested concentrations (fold 1.6–1.9). At concentrations of 1–10 µg/mL, *D. tertiolecta*, *I. galbana*, and *A. inequalis*, as well as *C. closterium* at 1 µg/mL, showed a beneficial effect on cell proliferation (BrdU incorporation fold increase), which was in line with the previous viability trends (Figure 1 and Figure 2). Interestingly, the BrdU results obtained for *Nostoc* spp. (fold change around 3 at 10 µg/mL) and *Xanthonema* spp. (fold change around 5 at 10 µg/mL), if interpreted in the context of proliferation, for which the BrdU assay was used, contradicted the results of the NRU and AB assays. We believe that this increase in DNA synthesis was due to slower cell cycle progression rather than higher proliferation, which resulted in higher BrdU signals due to the larger number of cells in the S-phase at 24–48 h of the experiment. This would also imply that, in both the control and, for example, *A. platensis*-treated cells, the first round of DNA synthesis was nearly completed before the addition of the BrdU; thus, slight fold differences were found. This hypothesis was investigated further in the Trypan blue exclusion test (Figure 3B).

The experiment was designed to examine the proliferation of ZEM2S cells in the presence of extracts in a 48-well microtiter plate at 72 h post-seeding in media with 1% FBS. The cells were harvested and counted using a hemocytometer in almost all wells, except for the wells in which the extracts of the tested algae/cyanobacteria did not give a satisfactory result in terms of the cell morphology (Figure 3B): *Nostoc* spp. and *P. galeata* at both 1 and 10 μg/mL, *Xanthonema* spp. and *I. galbana* at 10 μg/mL, and *D. tertiolecta* at 100 μg/mL. The cells showed the highest confluency and viability in the case of blue spirulina for all three concentrations, with even a slight increase in the cell numbers compared to the control. The extracts of *A. platensis* and *D. tertiolecta* were tolerated up to 10 μg/mL. Among the remaining few extracts (*I. galbana*, *C. closterium*, *N. gaditana*, and *A. inequalis)*, only *C. closterium* and *A. inequalis* at 1 μg/mL supported normal cell division and preserved the morphology. We monitored the morphological appearance of the cells daily for three days, as shown in Table 1. The cell morphology was compared to that of the control (untreated cells in 1% FBS media) using the following grading system: (+) unchanged, (+/−) changing but somewhat good, (−) morphology was lost.

### 3.3. Tertiary Screening of Selected Extracts in QM7 Myoblasts

**Figure 4:**
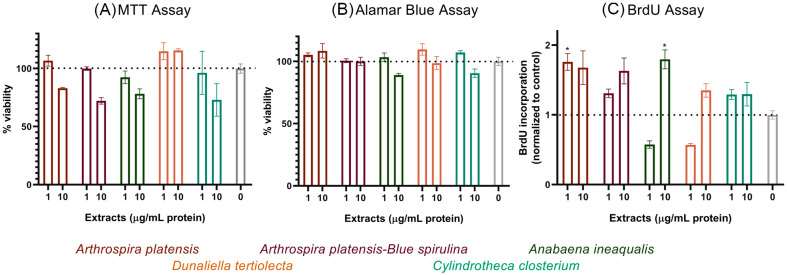
The effect of the selected extracts on QM7 cells. Cytotoxicity of five selected algal/cyanobacterial extracts was measured by the MTT assay (**A**) and Alamar Blue assay (**B**) after 72 h of treatment and normalized to the non-treated/control cells, which were at 100%. Cell proliferation was assessed by measuring DNA synthesis in a BrdU incorporation assay at 24–48 h (**C**). Each extract was tested at least twice; the graph presents the mean and SEM values of triplicate runs from one representative experiment. The legend contains the full names of the species, color-coded to match the columns. Statistically significant differences between the control and the treatment groups are labeled with * (*p* ≤ 0.05).

The following five extracts, which were consistent in all ZEM2S screening assays, were further analyzed at 1 and 10 μg/mL in QM7 myoblasts: *A. platensis* extract and in the form of blue spirulina, *A. inequalis*, *D. tertiolecta*, and *C. closterium.* As opposed to ZEM2S, the QM7 cells efficiently metabolized MTT, so the cell viability was analyzed by both the AB and MTT assays at 72 h post-seeding (Figure 4A,B). At 1 μg/mL, the extracts were not cytotoxic in both assays. However, some discrepancies were noted at the higher tested concentration for *A. platensis*, with MTT showing a decrease in cell viability to 70–80% (Figure 4A). As this decrease was not supported by the AB and BrdU incorporation analysis (Figure 4B,C), it is possible that it was measured due to cell detachment during sample processing. *D. tertiolecta* did not show any cytotoxic effects at 10 μg/mL, but this was not mirrored in the BrdU proliferation analysis (Figure 4C), where we could see cell cycle arrest, resulting in a lower fold increase at 1 μg/mL or some accumulation of cells in the S-phase of the cycle at 10 μg/mL. *A. platensis*, blue spirulina, and *C. closterium* were extracts with good alignment between the obtained results from the cytotoxicity and proliferation analysis (Figure 4).

### 3.4. Maintenance of ZEM2S and QM7 Cells in the Presence of Blue Spirulina

When considering the cell viability, proliferative capacity, and morphology at even the highest concentration of algae tested, the only extract that showed consistently good results throughout all tests was the blue spirulina extract. In addition, this preparation yielded an extract with a high protein concentration (11 mg/mL; Table 2), making scaling up possible without any complicated downstream concentration procedures, which can affect the quality of the extract. Therefore, this extract was selected for a multi-passage study in both QM7 and ZEM2S cells to investigate its ability to support cell growth for up to a month or through six passages.

Before the experiment, the cells were routinely maintained in media supplemented with 10% FBS and passaged every 3–4 days or when the confluency was 70–90%. On the day of the experiment, ZEM2S and QM7 cells were seeded in T-25 flasks in media with 1% FBS, either with or without 10 or 100 µg/mL of blue spirulina. In the first few passages, the cells were counted with TB to estimate the multiplication factor (Mf) by dividing the number of living cells at the time of passage by the number of living cells at the time of seeding. From the third or fourth passage, depending on the cell line, the cells were split at the ratio of 1/3. The pH of the spent media from both cell lines did not significantly alter during the initial monitoring period of up to four passages (Supplementary Table S1).

**Figure 5:**
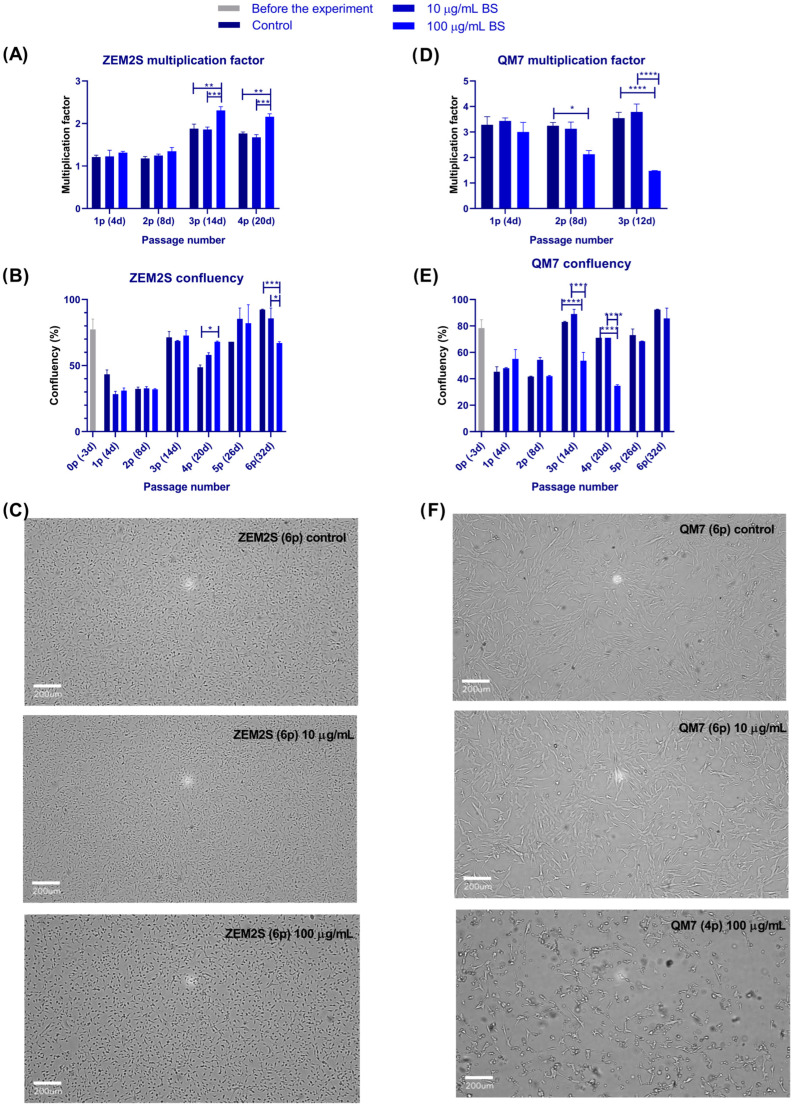
Maintenance of ZEM2S and QM7 cells in the presence of blue spirulina (BS) and 1% FBS. Before the experiment, cells were routinely maintained in media supplemented with 10% FBS and passaged every three days. Upon seeding in 1% FBS, with or without 10 or 100 µg/mL of BS, the cell morphology and confluency were determined by image-based analysis. Cells were passaged, counted with Trypan blue (1–4 passages), re-seeded, and kept in culture for up to 32 days (1–6 passages). The multiplication factor was determined by dividing the number of living cells at the time of passage by the number of living cells at the time of seeding. (**A**) Multiplication factor for ZEM2S cells at 1–4 passages in BS-supplemented media; (**B**) confluency of ZEM2S cells at 0–32 days; (**C**) morphology of ZEM2S cells at passage 6; (**D**) multiplication factor for QM7 cells at 1–3 passages in BS-supplemented media; (**E**) confluency of QM7 cells at 0–32 days; (**F**) morphology of QM7 cells at passages 6 and 4. Statistically significant differences between the control and the treatment groups are labeled with * (*p* ≤ 0.05), ** (*p* ≤ 0.01), *** (*p* ≤ 0.001), or **** (*p* ≤ 0.0001).

ZEM2S cell growth was impaired in the reduced-serum conditions, resulting in Mfs slightly above 1 and confluency of 30–40% on days four and eight (Figure 5A,B). As the cells were not multiplying fast enough, at the second passage (day 8), the cell seeding number was increased from 8E5 cells/flask to 1E6 cells/flask. From then on, ZEM2S were maintained in the culture for six days before passaging, including a media change every third day. With these changes, we achieved the minimal confluency requirement of 70% at the time of passage (3rd–6th passage; Figure 5B). There were no differences between the control and 1 µg/mL blue spirulina-treated cells; however, 100 µg/mL of blue spirulina significantly enhanced cell proliferation, with an average Mf of 2.2 at passages 3 and 4 (average control Mf was 1.8). After six passages and 32 days in culture with blue spirulina, the ZEM2S cells preserved their characteristic morphologies (Figure 5C). Still, at passage number six, changes were seen for the first time in the cells treated with 100 µg/mL. This drop in confluency was due to the presence of smaller, rounded cells (Figure 5C), which resulted in less area coverage, being read by the software as a drop in cell confluency.

The QM7 cells tolerated the reduced-serum conditions better than ZEM2S, as reflected in the higher Mfs, above three (Figure 5D), and the ability to adhere to the 4-day passaging schedule. These cells were seeded at 2E5 cells/T25 flask. It took the cells two passages to adapt to the new conditions, so, from passage 3, the cell number, morphology, and spreading were significantly better than at the beginning (70–80% vs. 40% confluency; Figure 5E). As with the ZEM2S cells, there were no noticeable differences in the tested period between the control and 10 µg/mL-treated cells (Figure 5D–F). In contrast to the ZEM2S cells, a higher concentration of blue spirulina, 100 µg/mL, was tolerated briefly by the QM7 cells, resulting in a significantly lower Mfs from passage 2, a drop in cell confluency at passage 4, and obvious changes in cell morphology (Figure 5D–F). The QM7 cells in the presence of 100 µg/mL were not propagated past passage number 4.

### 3.5. Biochemical Analysis of the Selected Extracts

We analyzed the biochemical compositions of the ten selected candidates to understand better the differences observed in the cell-based assays following the primary screening. The phenolic compounds, carbohydrates, and glucose concentrations and their antioxidant capacities were compared. Since we utilized the protein concentration to measure the amounts of extracts applied to each cell treatment, normalizing the above-mentioned parameters to the protein was the most suitable approach to facilitate straightforward correlation with the outcomes of the cell-based experiments. 

According to the results presented in Table 2, column B/A, the extract with the highest concentration of phenolic compounds was that of *Nostoc* spp., followed by *Xanthonema* spp. and *I. galbana*. These extracts did not perform as well as the others in the cell-based assays, especially *Nostoc* spp. and *Xanthonema* spp. (Figure 3). Carbohydrate-rich extracts were in the descending order of *Xanthonema* spp., *D. tertiolecta*, and *C. closterium*, with more than 15 mg of carbohydrate per 1 mg of protein in the extract (Table 2, column C/A). Among these three extracts, *D. tertiolecta* had the largest amount of glucose (60% of total carbohydrates), followed by *Xanthonema* spp. (23%) and *C. closterium* (13%) (Table 2, column D/A). Compared to the others, the *A. platensis* extract and its purified form (blue spirulina) were low in phenolic compounds and carbohydrate content. However, blue spirulina, which performed better in the previous assays, had 40 times more carbohydrates and glucose than the extract of *A. platensis*. The highest antioxidant capacity was measured in the extracts with the highest concentrations of phenolic compounds per mg of protein: blue spirulina, *Nostoc* spp., *P. galeata*, and *A. platensis* (Table 2, AC column). 

## 4. Discussion

This study thoroughly assessed the effects of twenty-six algal and cyanobacterial extracts on cells under reduced-serum conditions by examining the cell viability, proliferation, and morphology. Species were chosen to accommodate members from five phyla: *Cyanobacteria*, *Rhodophyta*, *Chlorophyta*, *Heterokontophyta*, and *Haptophyta*. Water extracts were prepared under mild alkaline conditions of pH 8.0 in low-molarity phosphate buffer to avoid any possible changes in the pH and osmolality of the cell media upon adding the extracts. The highest yield of proteins in the extract was obtained from the cyanobacteria, especially *A. platensis* and *P. galeata*, which is in line with the previously published data elaborated in a review study by Wang et al. (2021) [26]. Two animal cell lines were chosen: a fish cell line (ZEM2S) and a quail cell line (QM7). Both cell lines have a fibroblast-like appearance, but their origin differs; ZEM2S is of embryogenic origin, and QM7 is of muscle origin (ATCC). QM7 is, therefore, regarded as a myoblast (ATCC), which makes it interesting for cell-based meat technology. On the other hand, ZEM2S cells, originating from zebrafish, offer a sensitive in vitro assay system for the investigation of extracellular stimuli that promote and regulate cell differentiation [27]. Moreover, ZEM2S cells are a relevant model system for the development of cell-based seafood production and serum-free media for mammalian cell lines [28]. Consequently, experiments were initially performed on this specific cell line, followed by evaluating the algal extracts on QM7 cells. 

With the primary screening, we aimed to determine which extracts had a beneficial effect in promoting ZEM2S cell viability under the reduced-serum conditions of 1% (90% reduction compared to the maintenance cell media). The general trend observed was reduced cell viability at extract concentrations ≥ 10 μg/mL, especially at 50 and 100 μg/mL of extract proteins. However, the effects of the cyanobacterium *A. platensis* and *Chlorophyta D. tertiolecta* on cell viability were superior even at the higher tested concentrations. In the second round of screening, we studied the cell proliferation and concluded that five extracts with good alignment between the cytotoxicity and proliferation analyses should be studied in the QM7 cell culture: *A. platensis*, blue spirulina, *D. tertiolecta*, *A. inequalis*, and *C. closterium*. The extract that showed consistently good results in both cell cultures regarding the cell viability, proliferative capacity, and morphology at even the highest tested concentration was blue spirulina. This extract was selected for a multi-passage study in both cell types to investigate its ability to support cell growth for up to a month. In the tested period of 32 days, QM7 cells tolerated 10 µg/mL of blue spirulina in the reduced-serum conditions while preserving the ability to adhere to the 4-day passaging schedule. ZEM2S cells were cultured successfully in the presence of 10 and 100 µg/mL of blue spirulina; at some passages, cell proliferation was even better than in the control. However, ZEM2S proliferation in the reduced-serum media slowed down per se, and they adopted a 6-day passaging schedule. A recent study by Amirvaresi and Ovissipouron (2024) [29] on ZEM2S cells demonstrated that an algal protein hydrolysate supported the growth of ZEM2S cells at 1–100 µg/mL in the presence of 1% FBS for 72 h. They also noted that decreasing the serum concentration from 10% to 1% negatively affected ZEM2S proliferation and that adding the algal hydrolysate somewhat enhanced cell proliferation.

*D. tertiolecta* emerged as another interesting candidate, showing good cell tolerance at higher concentrations in both cell lines. It was not processed further through multiple passages because of the extract’s low protein concentration and the issue with cell proliferation arrest in ZEM2S cells at 100 µg/mL. The protein content for this microalgal species was determined to be at around 20% of the dry biomass [30], three times lower than the 60% reported for *A. platensis* [31]. Our extraction procedures resulted in more than a 10-fold difference in protein concentration between these two extracts, meaning that there is space for the implementation of new processing techniques to increase the extractability of *D. tertiolecta* proteins, such as supercritical fluid extraction and/or ultrahigh pressure [32]. On the other hand, the polyphenol content was low in the *D. tertiolecta* extract, but the concentration of glucose was superior when compared to the others, accounting for 60% of the total carbohydrates. If we perform calculations based on our data, with 10 µg of protein, there was 220 µg glucose supplementation per 1 mL of cell media. This positive ratio of glucose to protein might be the key to *D. tertiolecta*’s good performance in the cell-based assays presented here, as glucose is a significant energy source and one of the few components required in serum-free media [33]. For example, C2C12 mouse myoblasts almost died after two days in a glucose-free medium [34]. So, what induces ZEM2S proliferation arrest at 100 µg/mL of *D. tertiolecta* that is not found in blue spirulina? Algae from the genus *Dunaliella* are known for their extremely high content of carotenoids (up to 10–14% dry weight), which is commercially exploited [35,36]. Of course, carotenoids were found in *A. platensis*, but only at less than 0.5% of the dry biomass [37]. Although carotenoids offer beneficial antioxidant protection, high concentrations can induce cell cycle arrest in continuously proliferating cell lines [38].

QM7’s continued proliferation in the reduced-serum media was an interesting finding. For myoblasts of different origins, it is usually the opposite; they start to differentiate under reduced-serum conditions and cease cell proliferation [39]. In a recent study, the authors also used water-based cyanobacterial extracts from *A. inequalis* and Spirulina in serum-free media for the short-term cultivation of mouse C2C12 and QM7 myoblasts [18]. The results presented showed a drastic reduction in the cell proliferation rate in the serum-free media, regardless of the cyanobacterial extracts, compared to the 10% FBS conditions. The authors used only DMEM as their basal medium, while we used M199 as the basal medium, supplemented with TPB and 1% FBS. The better proliferation rates of the QM7 cells that we measured could be attributed to the nutrient-rich TPB, supplemented throughout the study at 10%, as recommended by the cell provider (ATCC). TPB is composed of four components, adjusted to a neutral pH and suitable for cell culture: tryptose (a mixture of amino acids and short peptides), dextrose, sodium chloride, and disodium phosphate (Sigma-Aldrich, cat. no. T8159). Therefore, in the future, we should reduce the concentration of TPB to determine whether QM7 cells can maintain their proliferative capacity. Similarly to our results, the authors reported that only lower concentrations of the tested cyanobacterial extracts were tolerated by the QM7 cells [18]. In an interesting study by Okamoto et al., 2019 [34], glucose and amino acids were extracted from different microalgal species. The most efficient extractions of glucose were from *Chlorococcum littorale* and *A. platensis*, whereas amino acids were efficiently extracted from *Chlorella vulgaris*. In line with our results, the authors reported that the supplementation of the C2C12 mouse myoblasts with these extracts significantly prevented cell death in glucose- and amino acid-free DMEM. One of the possible mechanisms for the growth-promoting activity of *A. platensis* extract, even at low concentrations, is the ability of C-phycocyanin, its major protein component, to upregulate various cyclin-dependent kinases and consequently enhance cell viability and proliferation, as demonstrated on dermal fibroblasts [40,41]. 

To the best of our knowledge, this is the first study that has comprehensively explored the effects of a broad spectrum of algal and cyanobacterial extracts on the proliferation, morphology, and viability of two animal cell lines relevant for cell-based seafood and meat production. Furthermore, a one-month multi-passage study was conducted on both cell lines, which is highly relevant in translating the obtained results to sustainable and long-term cell line cultivation at reduced-serum or serum-free conditions. There are only a few examples of multi-passage studies when it comes to searching for an appropriate serum substitute. In one case, a four-passage assay was performed to examine the effects of soybean meal and edible insect hydrolysates on the viability of pig muscle stem cells in 75% and 50% FBS-reduced media [42]. Another study to consider is an evaluation of a rapeseed protein isolate as a substitute for recombinant albumin in Beefy-9 serum-free media for bovine satellite cells, demonstrating enhanced cell growth and an improved doubling time over four passages [43].

In conclusion, the specific composition of the cyanobacterial and algal species is detrimental to their successful implementation in cell culture media formulations. Therefore, it is necessary to identify molecules with cell growth-promoting activity and components responsible for cell cytotoxicity. The amounts of basic cell nutrients and their extraction efficiency are other factors to consider, as extracts with large amounts of glucose and proteins performed better in the cell-based assays. Although our study did not prove the direct correlation between the total phenolic content and cytotoxicity, extracts rich in polyphenolic compounds, such as *Nostoc* spp. and *Xanthonema* spp., proved less compatible with the cells. On the other hand, the cells better tolerated extracts with a balance between polyphenolic compounds, glucose, and proteins. Of course, these are generalized conclusions, as a species-specific effect could be ascribed solely to their composition. 

## 5. Conclusions

In this study, we designed a comprehensive approach for the screening of extracts obtained from various algae and cyanobacteria, with the goal of selecting the most potent candidates for potential FBS replacement in cultivated meat technology. Extracts obtained from *Dunaliella tertiolecta* and *Arthrospira platensis* (blue spirulina) exhibited the highest tolerance in cell culture at reduced-serum conditions, considering short-term cultivation. *Cylindrotheca closterium*, *Isochrysis galbana*, and *Anabaena inequalis* were next in line, behind the primary screening hits, with promising potential for the future development of cell nutrients. On the other hand, only blue spirulina extract demonstrated the potential to stimulate cell growth over multiple passages during one month of cultivation. 

Developing such a screening platform and the biochemical characterization of the tested extracts should provide a means to establish a correlation between the extract composition and potency to support normal cell growth. This insight should enhance the creation of new cell media with significant promise for sustainable cell-based seafood and meat cultivation. Further studies should focus on identifying the key specific molecules from algae extracts responsible for maintaining and/or enhancing cell growth and proliferation. 

## Figures and Tables

**Table 1:** Morphological appearance of the cells in the presence of the extracts.

Cell Morphology												
Species	*A. platensis*			Blue Spirulina			*A. inequalis*		*Nostoc* sp.		*P. galeata*	
C (µg/mL)	1	10	50	1	10	100	1	10	1	10	1	10
24 h	+	+	+	+	+	+	+	+	+	−	+	+/−
48 h	+	+	+	+	+	+	+	+	+	−	+	+/−
72 h	+	+	+/−	+	+	+	+	+	−	−	−	−
Species	*D. tertiolecta*			*Xanthonema* spp.		*N. gaditana*		*C. closterium*		*I. galbana*		
C (µg/mL)	1	10	100	1	10	1	10	1	10	1	10	
24 h	+	+	+/−	+	+/−	+	+	+	+	+	−	
48 h	+	+	+/−	+	+/−	+	+	+	+	+	−	
72 h	+	+	−	+/−	−	+	+/−	+	+/−	+	−	

Cell morphology was compared to the control using the following grading system: (+) unchanged, (+/−) changing but somewhat good, (−) morphology was lost.

**Table 2:** Biochemical analysis of the extracts selected in the primary screening.

Extract	A: Protein (mg/mL)	B: Phenolic Compounds (mg/mL)	C: Carbohydrates (mg/mL)	D: Glucose (mg/mL)	B/A	C/A	D/A	AC (µmol Trolox/ mg Protein)
*Arthrospira platensis*	9.800	0.415	0.640	0.211	0.042	0.065	0.022	0.088
Blue Spirulina	11.000	0.519	29.000	9.511	0.047	2.636	0.865	0.201
*Anabaena inequalis*	1.064	0.064	1.140	0.053	0.060	1.072	0.050	0.171
*Nostoc species*	1.042	0.645	3.368	0.009	0.619	3.232	0.009	1.734
*Pseudanabaena galeata*	6.925	0.423	5.681	0.182	0.061	0.820	0.026	0.249
*Dunaliella tertiolecta*	0.600	0.020	22.450	13.210	0.034	37.410	22.020	0.369
*Xanthonema* species	0.230	0.046	11.930	2.761	0.201	51.860	12.000	0.797
*Nannochloropsis gaditana*	0.626	0.046	5.028	1.374	0.074	8.032	2.194	0.197
*Cylindrotheca closterium*	0.571	0.048	8.591	1.167	0.084	15.040	2.044	0.150
*Isochrysis galbana*	0.402	0.095	3.445	0.091	0.237	8.570	0.226	0.631

A three-color scale was used to label cells according to their value: red (low), yellow (middle), and green (high). AC—antioxidant capacity.

## Data Availability

The original contributions presented in the study are included in the article/Supplementary Materials, further inquiries can be directed to the corresponding author.

## Supplementary Materials

The following supporting information can be downloaded at: https://www.mdpi.com/article/10.3390/foods13233741/s1; Table S1: pH measurements in the spent cell media at the time of passage.

## References

[1] Benny A., Pandi K., Upadhyay R. (2022). Techniques, Challenges and Future Prospects for Cell-Based Meat. Food Sci. Biotechnol..

[2] Cai J., Wang S., Li Y., Dong S., Liang J., Liu Y., Li S. (2024). Industrialization Progress and Challenges of Cultivated Meat. J. Future Foods.

[3] Chen L., Guttieres D., Koenigsberg A., Barone P.W., Sinskey A.J., Springs S.L. (2022). Large-scale cultured meat production: Trends, challenges and promising biomanufacturing technologies. Biomaterials.

[4] Gaydhane M.K., Mahanta U., Sharma C.S., Khandelwal M., Ramakrishna S. (2018). Cultured meat: State of the art and future. Biomanuf. Rev..

[5] Bonny S.P., Gardner G.E., Pethick D.W., Hocquette J.-F. (2015). What is artificial meat and what does it mean for the future of the meat industry?. J. Integr. Agric..

[6] Treich N. (2021). Cultured Meat: Promises and Challenges. Environ. Resour. Econ..

[7] Kumar P., Chatli M.K., Mehta N., Singh P., Malav O.P., Verma A.K. (2017). Meat Analogues: Health Promising Sustainable Meat Substitutes. Crit. Rev. Food Sci. Nutr..

[8] Lanzoni D., Rebucci R., Formici G., Cheli F., Ragone G., Baldi A., Violini L., Sundaram T.S., Giromini C. (2024). Cultured Meat in the European Union: Legislative Context and Food Safety Issues. Curr. Res. Food Sci..

[9] Bhat Z.F., Kumar S., Fayaz H. (2015). In Vitro Meat Production: Challenges and Benefits over Conventional Meat Production. J. Integr. Agric..

[10] Tuomisto H.L., Teixeira De Mattos M.J. (2011). Environmental Impacts of Cultured Meat Production. Environ. Sci. Technol..

[11] Gottipamula S., Muttigi M.S., Kolkundkar U., Seetharam R.N. (2013). Serum-Free Media for the Production of Human Mesenchymal Stromal Cells: A Review. Cell Prolif..

[12] Stout A.J., Mirliani A.B., Rittenberg M.L., Shub M., White E.C., Yuen J.S.K., Kaplan D.L. (2022). Simple and Effective Serum-Free Medium for Sustained Expansion of Bovine Satellite Cells for Cell Cultured Meat. Commun. Biol..

[13] Hubalek S., Post M.J., Moutsatsou P. (2022). Towards Resource-Efficient and Cost-Efficient Cultured Meat. Curr. Opin. Food Sci..

[14] Ahmad A., Hassan S.W., Banat F. (2022). An Overview of Microalgae Biomass as a Sustainable Aquaculture Feed Ingredient: Food Security and Circular Economy. Bioengineered.

[15] Post M.J., Levenberg S., Kaplan D.L., Genovese N., Fu J., Bryant C.J., Negowetti N., Verzijden K., Moutsatsou P. (2020). Scientific, Sustainability and Regulatory Challenges of Cultured Meat. Nat. Food.

[16] Haraguchi Y., Okamoto Y., Shimizu T. (2022). A Circular Cell Culture System Using Microalgae and Mammalian Myoblasts for the Production of Sustainable Cultured Meat. Arch. Microbiol..

[17] Okamoto Y., Haraguchi Y., Yoshida A., Takahashi H., Yamanaka K., Sawamura N., Asahi T., Shimizu T. (2022). Proliferation and Differentiation of Primary Bovine Myoblasts Using Chlorella Vulgaris Extract for Sustainable Production of Cultured Meat. Biotechnol. Prog..

[18] Ghosh J., Haraguchi Y., Asahi T., Nakao Y., Shimizu T. (2023). Muscle Cell Proliferation Using Water-Soluble Extract from Nitrogen-Fixing Cyanobacteria *Anabaena* Sp. PCC 7120 for Sustainable Cultured Meat Production. Biochem. Biophys. Res. Commun..

[19] Jeong Y., Choi W.Y., Park A., Lee Y.J., Lee Y., Park G.H., Lee S.J., Lee W.K., Ryu Y.K., Kang D.H. (2021). Marine Cyanobacterium Spirulina Maxima as an Alternate to the Animal Cell Culture Medium Supplement. Sci. Rep..

[20] Machu L., Misurcova L., Ambrozova J.V., Orsavova J., Mlcek J., Sochor J., Jurikova T. (2015). Phenolic Content and Antioxidant Capacity in Algal Food Products. Molecules.

[21] Nielsen S.S. (2019). Correction to: Food Analysis Laboratory Manual.

[22] Subbiah V., Zhong B., Nawaz M.A., Barrow C.J., Dunshea F.R., Suleria H.A.R. (2021). Screening of Phenolic Compounds in Australian Grown Berries by LC-ESI-QTOF-MS/MS and Determination of Their Antioxidant Potential. Antioxidants.

[23] Mosmann T. (1983). Rapid Colorimetric Assay for Cellular Growth and Survival: Application to Proliferation and Cytotoxicity Assays. J. Immunol. Methods.

[24] Kumar P., Nagarajan A., Uchil P.D. (2018). Analysis of Cell Viability by the Alamarblue Assay. Cold Spring Harb. Protoc..

[25] Repetto G., del Peso A., Zurita J.L. (2008). Neutral Red Uptake Assay for the Estimation of Cell Viability/Cytotoxicity. Nat. Protoc..

[26] Wang Y., Tibbetts S.M., McGinn P.J. (2021). Microalgae as Sources of High-Quality Protein for Human Food and Protein Supplements. Foods.

[27] Ramos B.C.R., Moraes M.N.C.M., Poletini M.O., Lima L.H.R.G., Castrucci A.M.L. (2014). From Blue Light to Clock Genes in Zebrafish ZEM-2S Cells. PLoS ONE.

[28] Nikkhah A., Rohani A., Zarei M., Kulkarni A., Batarseh F.A., Blackstone N.T., Ovissipour R. (2023). Toward Sustainable Culture Media: Using Artificial Intelligence to Optimize Reduced-Serum Formulations for Cultivated Meat. Sci. Total Environ..

[29] Amirvaresi A., Ovissipour R. (2024). Assessment of Plant- and Microbial-Derived Protein Hydrolysates as Sustainable for Fetal Bovine Serum in Seafood Cell Culture Media. Fut. Foods.

[30] Brown M.R. (1991). The Amino-Acid and Sugar Composition of 16 Species of Microalgae Used in Mariculture. J. Exp. Mar. Bio. Ecol..

[31] Habib A.B., Parvin M., Huntington T.C., Hasan M.R. (2008). Review on Culture, Production and Use of Spirulina as Food for Humans and Feeds for Domestic Animals and Fish. FAO Fisheries and Aquaculture Circular.

[32] Amador-Luna V.M., Herrero M., Domínguez-Rodríguez G., Ibáñez E., Montero L. (2024). Enhancing the Bioactivity of Dunaliella Salina Extracts through Ultra-High Pressure Supercritical Fluid Extraction (UHP-SFE). Innov. Food Sci. Emerg. Technol..

[33] Ghasemi N., Bandehpour M., Ranjbari J. (2019). Optimization of Key Factors in Serum Free Medium for Production of Human Recombinant GM-CSF Using Response Surface Methodology. Iran. J. Pharm. Res..

[34] Okamoto Y., Haraguchi Y., Sawamura N., Asahi T., Shimizu T. (2020). Mammalian Cell Cultivation Using Nutrients Extracted from Microalgae. Biotechnol. Prog..

[35] Ye Z.W., Jiang J.G., Wu G.H. (2008). Biosynthesis and Regulation of Carotenoids in Dunaliella: Progresses and Prospects. Biotechnol. Adv..

[36] Pagels F., Guedes A.C., Jacob-Lopes E., Queiroz M.I., Maroneze M.M., Zepka L.Q. (2023). β-Carotene from Dunaliella: Production, Applications in Food/Feed, and Recent Advances. Handbook of Food and Feed from Microalgae.

[37] Marzorati S., Schievano A., Idà A., Verotta L. (2020). Carotenoids, Chlorophylls and Phycocyanin from Spirulina: Supercritical CO_2_ and Water Extraction Methods for Added Value Products Cascade. Green Chem..

[38] Jiří B., Lenka V., Josef S., Věra K. (2024). Exploring Carotenoids: Metabolism, Antioxidants, and Impacts on Human Health. J. Funct. Foods.

[39] Lawson M.A., Purslow P.P. (2000). Differentiation of Myoblasts in Serum-Free Media: Effects of Modified Media Are Cell Line-Specific. Cells Tissues Organs.

[40] Liu P., Choi J.W., Lee M.K., Choi Y.H., Nam T.J. (2019). Wound Healing Potential of Spirulina Protein on CCD-986sk Cells. Mar. Drugs.

[41] Madhyastha H.K., Radha K.S., Nakajima Y., Omura S., Maruyama M. (2008). UPA Dependent and Independent Mechanisms of Wound Healing by C-Phycocyanin. J. Cell Mol. Med..

[42] Kim C.H., Lee H.J., Jung D.Y., Kim M., Jung H.Y., Hong H., Choi Y.S., Yong H.I., Jo C. (2023). Evaluation of Fermented Soybean Meal and Edible Insect Hydrolysates as Potential Serum Replacement in Pig Muscle Stem Cell Culture. Food Biosci..

[43] Stout A.J., Rittenberg M.L., Shub M., Saad M.K., Mirliani A.B., Dolgin J., Kaplan D.L. (2023). A Beefy-R Culture Medium: Replacing Albumin with Rapeseed Protein Isolates. Biomaterials.

