# Supplementary material for: Screening Algal and Cyanobacterial Extracts to Identify Potential Substitutes for Fetal Bovine Serum in Cellular Meat Cultivation
Source: Foods. 2024 Nov 22;13(23):3741. doi: 10.3390/foods13233741 (PMC11640053; doi:10.3390/foods13233741)
Supplement: Supplementary file 1 [file foods-13-03741-s001.zip › foods-3268120-supplementary.pdf]

Supplementary information

Screening algal and cyanobacterial extracts to identify potential substitutes for fetal bovine serum in cellular meat cultivation

Nikolina Sibinčić [1], Maja Krstić Ristivojević [2], Nikola Gligorijević [3], Luka Veličković [2], Katarina Ćulafić [2], Zorana Jovanović [2], Aleksandar Ivanov [2], Lora Tubić [2], Carole Vialleix [4], Thibaut Michel [4], Tatjana Srdić Rajić [5], Milan Nikolić [2], Marija Stojadinović [2,*], Simeon Minić [2]

[1] Innovative Centre, University of Belgrade – Faculty of Chemistry, Belgrade, Serbia
[2] Department of Biochemistry & Center of Excellence for Molecular Food Sciences, University of Belgrade – Faculty of Chemistry, Belgrade, Serbia
[3] Department of Chemistry, Institute of Chemistry, Technology, and Metallurgy, National Institute of the Republic of Serbia, University of Belgrade, Belgrade, Serbia
[4] GreenSea, Mèze, France
[5] Institute for Oncology and Radiology of Serbia, Belgrade, Serbia
* Correspondence: mstojadinovic@chem.bg.ac.rs

Results

Table S1. pH measurements in the spent cell media at the time of passage (p).

**Cell line:**	**ZEM2S**			**QM7**		
**Blue Spirulina concentration:**	**0 µg/mL**	**10 µg/mL**	**100 µg/mL**	**0 µg/mL**	**10 µg/mL**	**100 µg/mL**
**1 p**	7.40	7.40	7.40	7.25	7.31	7.21
**2 p**	7.45	7.58	7.59	7.28	7.30	7.12
**3 p**	7.36	7.39	7.40	7.23	7.30	7.05
**4 p**	7.14	7.19	7.21			
